# The effect of interactions between BMI and sustained depressive symptoms on knee osteoarthritis over 4 years: data from the osteoarthritis initiative

**DOI:** 10.1186/s12891-023-06132-3

**Published:** 2023-01-12

**Authors:** Gabby B. Joseph, Charles E. McCulloch, Michael C. Nevitt, John Lynch, Nancy E. Lane, Valentina Pedoia, Sharmila Majumdar, Thomas M. Link

**Affiliations:** 1 Department of Radiology and Biomedical Imaging, University of California, 185 Berry St, Suite 350, San Francisco, CA 94158 USA; 2 Department of Epidemiology and Biostatistics, University of California, San Francisco, USA; 3 Department of Rheumatology, University of California, Davis, USA

**Keywords:** Depression, Obesity, MRI, Cartilage T_2_, JSN

## Abstract

**Background:**

To assess the compound effects of BMI and sustained depressive symptoms on changes in knee structure, cartilage composition, and knee pain over 4 years using statistical interaction analyses.

**Methods:**

One thousand eight hundred forty-four individuals from the Osteoarthritis Initiative Database were analyzed at baseline and 4-year follow-up. Individuals were categorized according to their BMI and presence of depressive symptoms (based on the Center for Epidemiological Studies Depression Scale (threshold≥16)) at baseline and 4-year follow-up. 3 T MRI was used to quantify knee cartilage T_2_ over 4 years, while radiographs were used to assess joint space narrowing (JSN). Mixed effects models examined the effect of BMI-depressive symptoms interactions on outcomes of cartilage T_2_, JSN, and knee pain over 4-years.

**Results:**

The BMI-depressive symptoms interaction was significantly associated with knee pain (*p* < 0.001) changes over 4 years, but not with changes in cartilage T_2_ (*p* = 0.27). In women, the BMI-depressive symptoms interaction was significantly associated with JSN (*p* = 0.01). In a group-based analysis, participants with obesity and depression had significantly greater 4-year changes in knee pain (coeff._(obesity + depression vs. no_obesity + no_depression)_ = 4.09, 95%CI = 3.60–4.58, *p* < 0.001), JSN (coeff. = 0.60, 95%CI = 0.44–0.77, *p* < 0.001), and cartilage T_2_ (coeff. = 1.09, 95%CI = 0.68–1.49, *p* < 0.001) than participants without depression and normal BMI.

**Conclusions:**

The *compound* effects of obesity and depression have greater impact on knee pain and JSN progression compared to what would be expected based on their *individual* effects.

## Introduction

Osteoarthritis (OA) is a multi-factorial, degenerative joint disease, affecting 10.5% of the US population (from The Institute of Health Metrics Evaluation Global Burden of Disease Tool), causing joint pain and chronic disability [1]. Obesity, which is prevalent in approximately 39.8% of US adults (data from 2015/2016 [2]), and depressive symptoms found in 8.4% of US adults [3]) are two comorbid conditions that are individually associated with OA. While many studies have assessed the *individual* effects of obesity and depression on OA [4–11], few have assessed the *compound* effects of these risk factors on knee joint structure, cartilage structure, and symptoms [12].

Obesity and depressive symptoms are two potentially modifiable risk factors for OA [4, 5]. Obesity is associated with increases in knee pain and disability [6], joint space narrowing [7], prevalence of knee cartilage lesions [8], and cartilage biochemical degeneration, which can be analyzed with MRI based T_2_ relaxation time measurements that are sensitive to alterations in collagen structure and water content [11]. Moreover, every 5 kg of weight gain increases the risk for OA by 36% (studied in in women aged 45–64, [5]). Depressive symptoms in adults are also associated with increases in joint pain [9] and disability [10], while patterns of osteophyte progression and JSN progression were not found significantly different between depressed and non-depressed individuals over 4 years [13]. However, individuals with mild or moderate-to-severe depression are two or three times more likely to develop knee OA than those without depression [14].

While previous studies have reported associations of both excess body mass and depressive symptoms on symptomatic OA, the knowledge gap on the compound effects of these risk factors on longitudinal changes in cartilage biochemical composition (i.e., MRI knee cartilage T_2_) remains to be investigated. Understanding the co-morbid effects of both obesity and depression on OA outcomes could guide patient-specific treatments that concurrently target obesity and depression with an overall goal to slow OA progression. Thus, the purpose of this study was to assess the compound effects of BMI and sustained depressive symptoms on changes in knee structure, cartilage composition, and knee pain over 4 years using statistical interaction analyses. The hypothesis of this study is that individuals with sustained depression have a more progressive course of structural OA and that presence of obesity amplifies this progressive course, more than expected for individual effects alone.

## Materials and methods

### Subject selection

This study utilized data from the Osteoarthritis Initiative (OAI; https://nda.nih.gov/oai) [15], a multi-center, longitudinal study of individuals aged 45–79 years at enrollment. The OAI dataset includes MRI and radiographic knee images of participants over 8 years. The study protocol, amendments, and informed consent documentation were reviewed and approved by the local institutional review boards of all participating centers (University of Maryland School of Medicine, Ohio State University, University of Pittsburgh, Memorial Hospital of Rhode Island). In addition, all methods were performed in accordance with the relevant guidelines and regulations the Human Research Protection Program (HRPP) at UC San Francisco.

**Fig. 1:**
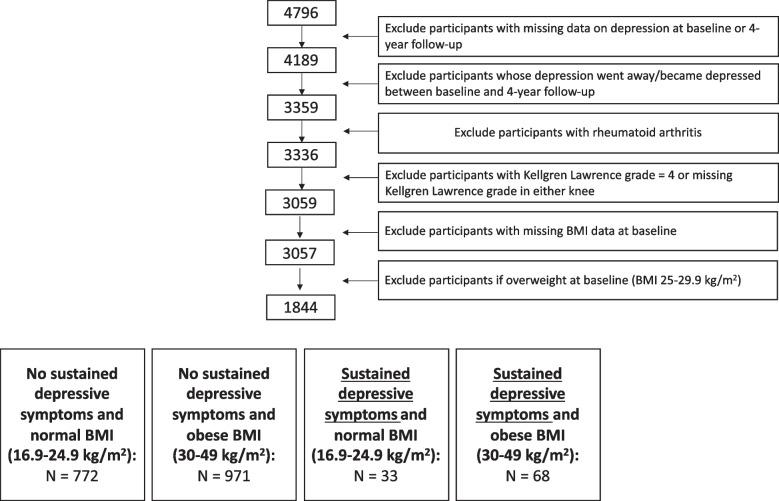
Participant Selection from the OAI. Note that sustained depressive symptoms were defined based on The Center for Epidemiological Studies Depression Scale (threshold ≥16) at the baseline and 4-year follow-up visit [13, 16]

The present study analyzed participants enrolled in the OAI with the following inclusion criteria: (i) available data on the Center for Epidemiological Studies Depression Scale at the baseline and 4-year follow-up visit, (ii) a baseline Kellgren Lawrence score (KL) ≤ 3 in the right or left knee, (iii) available body mass index (BMI) data at baseline and (iv) either normal BMI (16.9–24.9 kg/m^2^) or obese BMI (30–49 kg/m^2^) at baseline. The overweight group was excluded to better investigate the effects of obesity in comparison to a normal BMI control cohort (16.9–24.9 kg/m^2^). Participants were excluded if their depression symptoms no longer met the threshold between baseline and 4-year follow-up, or participants became depressed (detailed description below) between baseline and 4-year follow-up. Participants with rheumatoid arthritis were also excluded. Based on these criteria, a total of 1844 participants (mean BMI: 28.8 ± 5.90 kg/m^2^) were included in this study **(**Fig. 1**)** and were categorized into 4 groups: no sustained depressive symptoms (defined below) and normal BMI (16.9–24.9 kg/m^2^), *n* = 772; no sustained depressive symptoms and obese BMI (30–49 kg/m^2^), *n* = 971; sustained depressive symptoms and normal BMI (16.9–24.9 kg/m^2^), *n* = 33; and sustained depressive symptoms and obese BMI (30–49 kg/m^2^), *n* = 68.

### Depressive symptoms

Depressive symptoms were assessed using the Center for Epidemiological Studies Depression Scale (CES-D) [17] (threshold ≥16) at the baseline and 4-year follow-up visit based on previous studies [13]. The CES-D is a 20-item questionnaire that asks individuals how often they experience symptoms associated with depression. This questionnaire has good sensitivity and specificity as well as a high internal consistency [18]. A threshold of ≥16 is often recommended as a cutoff when for screening for “clinical depression” [13] based on published studies [13, 16]. Participants with sustained high level of depressive symptoms were defined as those a CES-D score of ≥16 at baseline and 4-year follow-up, while participants without sustained depressive symptoms had a CES-D score of < 16 at baseline and 4-year follow-up. Participants with depressive symptoms that were not sustained between baseline (CES-D ≥ 16) and 4-year follow-up (CES-D < 16) or became depressed between baseline (CES-D < 16) and 4-year follow-up (CES-D ≥ 16) were excluded to focus the analysis on participants with or without depressive symptoms at both timepoints.

### Additional clinical questionnaires

Knee pain was assessed using the *WOMAC (Western Ontario McMaster Universities Osteoarthritis) Index,* a standard questionnaire used to evaluate symptoms related to knee OA, including pain, limited function and stiffness [19]. This questionnaire has three subscales (pain (range: 0 to 20), stiffness (range: 0 to 8), and physical function (range: 0 to 68)) and has been utilized in a number of previous OA studies [20, 21]. The current study focuses on the WOMAC pain subscore; higher scores indicate worse pain.

The participants’ physical activity levels were determined using a *Physical Activity Scale for the Elderly (PASE*) with a range of 0 to 400. This is a well-established, reliable, validated questionnaire that has been used to measure physical activity in individuals of similar age to those investigated in the current study [22–25]. The areas of assessment are activities of occupation, household, and leisure activities over a 1 week period.

### Radiographs

Standardized bilateral standing posterior-anterior fixed flexion knee radiographs were acquired in all participants in the OAI. For eligibility and to assess baseline disease burden, knee Kellgren Lawrence (KL) gradings [26] were performed at baseline with a score ranging from 0 (none) to 4 (severe). A KL grade of 0 represents definite absence of radiographic changes of OA; grade 1 represents: doubtful joint space narrowing (JSN) and possible osteophytic lipping; grade 2 represents definite osteophytes and possible JSN; grade 3 represents moderate multiple osteophytes, definite JSN and some sclerosis and possible deformity of bone ends; grade 4 represents: large osteophytes, marked JSN, severe sclerosis and definite deformity of bone ends. In addition, JSN (maximum score of the medial and lateral joint sides in each knee) was assessed longitudinally from baseline to 2- and 4-year follow-up [27] based on the OARSI grading scale.

### MR imaging acquisition and analyzed parameters

#### MR imaging acquisition

MR imaging was performed using 3 T MRI scanners (Trio, Siemens, Erlangen, Germany) at four centers (Ohio State University in Columbus, University of Maryland in Baltimore, University of Pittsburgh and Brown University in Rhode Island) as part of the imaging OAI protocol. The following sequence of the right knee were analyzed in this study: sagittal 2D multi-echo (ME) spin-echo (SE) sequences for T_2_ quantification. The imaging parameters for the MESE T_2_ mapping sequence were: TR = 2700 ms, 7 TEs = 10, 20, 30, 40, 50, 60 and 70 ms, in-plane spatial resolution of 0.313 mm × 0.446 mm (0.313 mm × 0.313 mm after reconstruction), slice thickness of 3.0 mm, and 0.5 mm gap. These scanning parameters were optimized based on the OAI MR imaging protocol; additional details on image acquisition parameters have been previously published [15].

#### Cartilage T_2_

MRI cartilage T_2_ measurements quantify the composition of the cartilage extracellular matrix, which includes collagen integrity and orientation, as well as water content. Cartilage T_2_ measurements of the right knees were quantified at baseline, 2, and 4 years in six regions (medial and lateral tibia, medial and lateral femur, trochlea, and patella). A deep learning-based algorithm with 2D U-Net convolutional neural networks, with high efficacy and precision, was utilized for automatic cartilage segmentation and T_2_ quantification as previously described [28, 29]. Briefly, the dataset was randomly split to training, validation, and test sets (65:25:10) and 3D V-Net architecture was used for segmentation. Although the OAI dataset provided images with 7 echoes (TE = 10, 20, 30, 40, 50, 60, 70 ms) for T_2_ quantification, the first echo (TE = 10 ms) was not included in the T_2_ fitting procedure in order to reduce potential errors resulting from stimulated echoes, and a noise-corrected algorithm was implemented [30, 31]. Average T_2_ values for each region were computed and analyzed in this study.

### Statistical analysis

Descriptive statistics were performed using a SAS Studio (version 3.8, SAS Institute Inc., Cary, NC, USA) macro program called “Tablen” [32]. Differences in continuous parameters between groups (i.e., age, BMI) were assessed using Kruskal Wallis tests, and differences in categorical parameters between groups (i.e., sex and race) were assessed using Chi-squared tests.

The primary statistical analyses were performed using STATA version 16 software (StataCorp LP, College Station, TX, USA) with significance set to *p* < 0.05. Two types of mixed effects models were performed (described below).

The first set of mixed models *were interaction analyses* to assess whether having both sustained depressive symptoms and obese BMI had a greater effect on knee outcomes (JSN, cartilage T_2_, and knee pain) over and above the additive effects of each predictor. The mixed models included a test for statistical interaction between BMI (normal/obese) and sustained depressive symptoms over 4-years (yes/no). All outcomes were treated as continuous variables. First, a model with a triple interaction was coded (interaction between BMI (normal/obese), depression (yes/no) and by time (years), in order to capture BMI-depressive symptoms interactions in the change in the outcome over time. If this interaction was not significant, the model was further simplified by including three double interactions (depression-BMI, depression-time, BMI-time). The interactions reported in this study are between BMI (normal/obese) and sustained depression (yes/no) as none of the interactions for longitudinal change were statistically significant (*p* > 0.05). JSN and cartilage T_2_ outcomes were analyzed at baseline, 2 and 4 years, while knee pain outcomes were analyzed annually over 4 years. A random effect for both person and knee were modelled for all outcomes except cartilage T_2_. A random effect for only person was modelled for cartilage T_2_ outcomes since cartilage T_2_ measurements were only obtained in the right knee in the OAI, and thus accounting for two knees was not needed.

The second set of mixed models (that did not include an estimate for and test for an interaction) *were group-based analyses* that investigated the overall differences in outcomes (JSN, cartilage T_2_, and knee pain) over all timepoints between participants subdivided into four groups based on baseline BMI (normal/obese) and sustained depression over 4 years (yes/no). The four groups were: no sustained depression and normal BMI (16.9–24.9 kg/m^2^), no sustained depression and obese BMI (30–49 kg/m^2^), sustained depression and normal BMI (16.9–24.9 kg/m^2^), sustained depression and obese BMI (30–49 kg/m^2^). The coefficients (which represent the difference in outcomes between each group and the reference group averaged over all timepoints) and *p*-values were derived from these model outputs. These analyses are described as *“group-based*” in the results section.

All mixed effects models were adjusted for age, sex, race, and PASE score. All assumptions for linear mixed models including a normal distribution and independent errors were met.

The outcome variables were designated as primary or exploratory to address potential issues stemming from multiple testing [33]. For cartilage T_2_, the primary analyses focused on the average of all regions (medial and lateral tibia, medial and lateral femur, trochlea, and patella). For JSN, the maximum score of the medial and lateral joint sides in each knee was assessed. For the WOMAC score, only the pain subscale was assessed. The remaining outcomes were designated as exploratory.

As a sensitivity analysis, an interaction between BMI-depression-sex was added to each model to assess whether the effects of BMI and depressive symptoms on outcomes differed by sex. Another sensitivity analysis was performed in participants with KL 0 or 1 in both knees to assess participants without radiographic evidence of OA in either knee. The first sensitivity analysis was included to assess whether the results of the main analyses differed by sex; the second sensitivity analysis was included to assess whether the results held true in participants without radiographic OA.

## Results

### Participant characteristics

One thousand eight hundred forty-four participants were included in this study; of those 68 had sustained depressive symptoms and obese BMI (30–49 kg/m^2^), 33 had sustained depressive symptoms and normal BMI (16.9–24.9 kg/m^2^), 971 had no sustained depressive symptoms and obese BMI (30–49 kg/m^2^) and 772 had no sustained depressive symptoms and normal BMI (16.9–24.9 kg/m^2^). The participant characteristics are listed in Table 1**.** The average BMI in participants with depressive symptoms and obesity (35.0 ± 3.58 kg/m^2^) was greater than that in the other groups **(**Table 1**)** including participants with no depressive symptoms and obese BMI (33.4 ± 2.96 kg/m^2^, *p* < 0.001). Participants with no depressive symptoms and normal BMI were the eldest (61.2 ± 9.29 years) compared the other groups (age range 58.9–60.4 years, *p* = 0.002). There were significant differences in the PASE score between groups (*p* = 0.009), with the highest PASE score in participants without depressive symptoms and normal BMI (169.0 ± 77.46). There were statistically significant differences in the distribution of race (*p* < 0.001) and KL grade between groups (*p* < 0.001 for both the right and left knees) as shown in Table 1.

**Table 1:** Participant characteristics at the baseline timepoint. Abbreviations: KL: Kellgren Lawrence, PASE: physical activity scale for the elderly; CES-D: the Center for Epidemiological Studies Depression; JSNmax: maximum joint space narrowing score**.** Note: cartilage T_2_ sequences were only acquired in the right knee in the OAI

	No Depression & Normal BMI (*N* = 772)	Depression & Normal BMI (*N* = 33)	No Depression & Obese (*N* = 971)	Depression & Obese (*N* = 68)	Total (*N* = 1844)	***P***-value
**Age (years)**						0.0021^1^
Mean (SD)	61.2 (9.29)	58.9 (8.64)	60.1 (8.49)	57.5 (8.30)	60.4 (8.86)	
**BMI (kg/m**^**2**^**)**						< 0.0001^1^
Mean (SD)	22.8 (1.55)	22.0 (2.19)	33.4 (2.96)	35.0 (3.58)	28.8 (5.90)	
**Sex, n (%)**						< 0.0001^2^
Male	232 (30.1%)	8 (24.2%)	399 (41.1%)	16 (23.5%)	655 (35.5%)	
Female	540 (69.9%)	25 (75.8%)	572 (58.9%)	52 (76.5%)	1189 (64.5%)	
**CES-D scale**						< 0.0001^1^
Mean (SD)	4.0 (3.70)	24.9 (9.25)	4.7 (3.94)	23.9 (7.86)	5.5 (6.18)	
**WOMAC pain right**						< 0.0001^1^
Mean (SD)	1.3 (2.05)	1.9 (3.27)	2.3 (2.91)	5.4 (4.61)	2.0 (2.81)	
**WOMAC pain left**						< 0.0001^1^
Mean (SD)	1.2 (2.29)	1.9 (3.05)	2.2 (3.20)	5.9 (5.13)	1.9 (3.09)	
**Race, n (%)**						< 0.0001^2^
0 – Other-non-white	4 (0.5%)	0 (0.0%)	14 (1.4%)	1 (1.5%)	19 (1.0%)	
1 – White or Caucasian	710 (92.1%)	27 (81.8%)	724 (74.6%)	42 (61.8%)	1503 (81.6%)	
2 – Black or African American	41 (5.3%)	5 (15.2%)	230 (23.7%)	25 (36.8%)	301 (16.3%)	
3 - Asian	16 (2.1%)	1 (3.0%)	3 (0.3%)	0 (0.0%)	20 (1.1%)	
Missing	1	0	0	0	1	
**KL grade right, n (%)**						< 0.0001^2^
0	429 (55.6%)	18 (54.5%)	282 (29.0%)	14 (20.6%)	743 (40.3%)	
1	137 (17.7%)	3 (9.1%)	183 (18.8%)	13 (19.1%)	336 (18.2%)	
2	146 (18.9%)	9 (27.3%)	335 (34.5%)	26 (38.2%)	516 (28.0%)	
3	60 (7.8%)	3 (9.1%)	171 (17.6%)	15 (22.1%)	249 (13.5%)	
**KL grade left, n (%)**						< 0.0001^2^
0	445 (57.6%)	20 (60.6%)	307 (31.6%)	15 (22.1%)	787 (42.7%)	
1	135 (17.5%)	3 (9.1%)	187 (19.3%)	9 (13.2%)	334 (18.1%)	
2	137 (17.7%)	7 (21.2%)	320 (33.0%)	28 (41.2%)	492 (26.7%)	
3	55 (7.1%)	3 (9.1%)	157 (16.2%)	16 (23.5%)	231 (12.5%)	
**PASE**						0.0099^1^
Mean (SD)	169.0 (77.46)	142.0 (83.50)	160.7 (85.64)	156.3 (91.98)	163.7 (82.63)	
**Mean cartilage T**_**2**_ **right (ms)**						0.0003^1^
Mean (SD)	33.1 (1.95)	33.3 (2.39)	33.3 (1.98)	34.0 (2.17)	33.3 (1.99	
**JSNmax right**						< 0.001^1^
Mean (SD)	0.4 (0.62)	0.3 (0.65)	0.7 (0.76)	0.9 (0.76)	0.5 (0.72)	
**JSNmax left **						< 0.001^1^
Mean (SD)	0.3 (0.60)	0.3 (0.65)	0.6 (0.75)	0.8 (0.80)	0.5 (0.71)	

^1^Kruskal-Wallis *p*-value; ^2^Chi-Square *p*-value;

### Joint space narrowing (JSN)

**Fig. 2:**
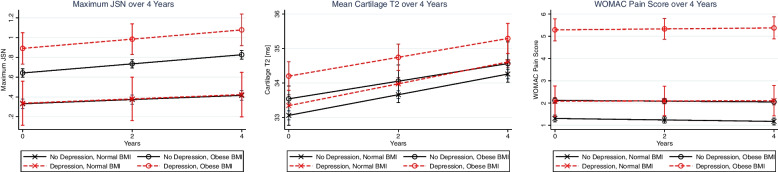
The graphs (derived from the interaction models) illustrate the longitudinal changes in maximum JSN [range 0 to 3], cartilage T_2_ [in ms], and WOMAC pain score [range 0 to 20] over 4 years. The depression-BMI interactions were statistically significant with WOMAC pain (*p* < 0.001). The *p*-value for the depression-BMI interaction on JSN was *p* = 0.08; the interaction was not significant for cartilage T_2_ (*p* = 0.27). The figure illustrates that the compound effects of obesity and depression on OA are greater than their individual effects: in all three outcomes, the difference between the normal BMI groups (denoted by X) is less than the obese groups (denoted by O). Thus, the effect of depression is stronger in the obese groups than the normal weight groups

The test for interaction **(**Table 2**)** between sustained depressive symptoms (yes/no) and BMI (normal/obese) on maximum JSN had *p* = 0.08, with the fitted model illustrated in Fig. 2*.* From the group-based analysis, over 4 years, maximum JSN was significantly greater in participants with *depressive symptoms and an obese BMI* compared to the other groups (Coeff. _over 4 years, *no depression and normal BMI*_ = 0.60, *p* < 0.001, 95%CI = 0.44–0.77; Coeff. _over 4 years, no *depression and obese BMI*_ = 0.25, *p* = 0.002, 95%CI = 0.09–0.41; Coeff. _over 4 years, *depression and normal BMI*_ = 0.60, *p* < 0.001, 95%CI = 0.33–0.87. The rates of change in JSN over 4 years between the four participant groups were not significantly different (*p* = 0.52). Table 2 lists the comparisons in JSN over 4 years between all groups compared to a reference group of no depressive symptoms and normal BMI.

**Table 2:** Interactions between BMI (normal/obese) and sustained depression over 4 years (yes/no) and outcomes (WOMAC pain, JSN, cartilage T_2_). An additional interaction between BMI-sustained depression-sex was included to test for sex differences. If significant, the analysis was subdivided by sex. All mixed effects models were adjusted for age, sex, BMI, and race

	WOMAC Pain	JSN	Cartilage T_**2**_
***P*** **value for the interaction between BMI and sustained depression**	**< 0.001**	0.08	0.27
***P*** **value for the interaction between BMI and sustained depression in participants with KL 0/1 in both knees at baseline**	**< 0.001**	**0.02**	0.25
***P*** **value for the interaction between BMI and sustained depression and sex (male/female)**	**0.02**	**0.03**	0.39
***P*** **value for the interaction between BMI and sustained depression) in males**	0.33	0.35	*
***P*** **value for the interaction between BMI and sustained depression in females**	**< 0.001**	**0.01**	*

* Note analysis this analysis was not subdivided by males/females since the interaction BMI and sustained depression and sex was not significant

### WOMAC pain

In the mixed effects regression model with WOMAC pain as an outcome, the interaction between BMI (normal/obese) and sustained depressive symptoms (yes/no) was statistically significant (*p* < 0.001) as shown in Table 2. An illustration of the BMI-depression interaction on WOMAC pain is presented in Fig. 2. From the group-based analysis, over 4 years, the WOMAC pain score was significantly greater in participants with depressive symptoms and obese BMI compared to the other groups (Coeff. _over 4 years, *no depression and normal BMI*_ = 4.09, *p* < 0.001, 95%CI = 3.60–4.58; Coeff. _over 4 years, no *depression and obese BMI*_ = 3.24, *p* < 0.001, 95%CI = 2.76–3.73; Coeff. _over 4 years, *depression and normal BMI*_ = 3.23, *p* < 0.001, 95%CI = 2.42–4.05. The rates of change in WOMAC pain over 4-years between the four participant groups were not significantly different (*p* = 0.98). Table 3 lists the comparisons in WOMAC pain over 4 years between all groups compared to a reference group of no depression and normal BMI.

**Table 3:** The associations of BMI/Depression group with WOMAC Pain, maximum JSN and cartilage T_2_ [ms]. All mixed effects models were adjusted for age, sex, BMI, and race. Abbreviations: SE: standard error; CI: confidence interval; Coeff: coefficient

	Beta Coeff.	SE	95% CI	***P*** value
**Maximum JSN**				
No Depression/Normal BMI _(*N* = 772)_	Reference			
No Depression/Obese BMI _(*N* = 971)_	0.35	0.03	0.29–0.42	**< 0.001**
Depression/Normal BMI _(*N* = 33)_	0.01	0.11	−0.21-0.23	0.90
Depression/Obese BMI _(*N* = 68)_	0.60	0.08	0.44–0.77	**< 0.001**
**WOMAC Pain**				
No Depression/Normal BMI _(*N* = 772)_	Reference			
No Depression/Obese BMI _(*N* = 971)_	0.84	0.09	0.65–1.03	**< 0.001**
Depression/Normal BMI _(*N* = 33)_	0.85	0.34	0.17–1.543	**0.01**
Depression/Obese BMI _(*N* = 68)_	4.09	0.25	3.60–4.58	**< 0.001**
**Cartilage T**_**2**_				
No Depression/Normal BMI _(*N* = 772)_	Reference			
No Depression/Obese BMI _(*N* = 971)_	0.39	0.08	0.24–0.55	**< 0.001**
Depression/Normal BMI _(*N* =33)_	0.31	0.28	−0.24-0.87	0.27
Depression/Obese BMI _(*N* = 68)_	1.09	0.04	0.68–1.49	**< 0.001**

### Cartilage T_2_ measurements

The depression-BMI interaction **(**Table 2**)** with average cartilage T_2_ as an outcome was not statistically significant **(**Table 2**,** *p* = 0.27). Average cartilage T_2_ increased over time in all four groups; however, the rates of change between the four groups were not significantly different (Fig. 1**,** *p* = 0.73). From the group-based analysis, over 4 years, the T_2_ was significantly greater in participants with depressive symptoms and obese BMI compared to the other groups (Coeff. _over 4 years, *no depression and normal BMI*_ = 1.09, *p* < 0.001, 95%CI = 0.68–1.49; Coeff*.*_over 4 years, no *depression and obese BMI*_ = 0.69, *p* = 0.001, 95%CI = 0.29–1.08; Coeff*.*_over 4 years, *depression and normal BMI*_ = 0.77, *p* = 0.02, 95%CI = 0.11–1.44. These results demonstrate that individuals with depressive symptoms and obesity had significant elevations in T_2_ (over all timepoints) compared to all other groups including individuals without depressive symptoms and without obesity. Table 2 lists the comparisons in cartilage T_2_ over 4 years between all groups compared to a reference group of no depression and normal BMI. To further examine the differences in cartilage T_2_ between groups (since the depression-BMI interaction was not statistically significant), an additional exploratory analysis was performed.

### Sensitivity analysis: sex differences

**Fig. 3:**
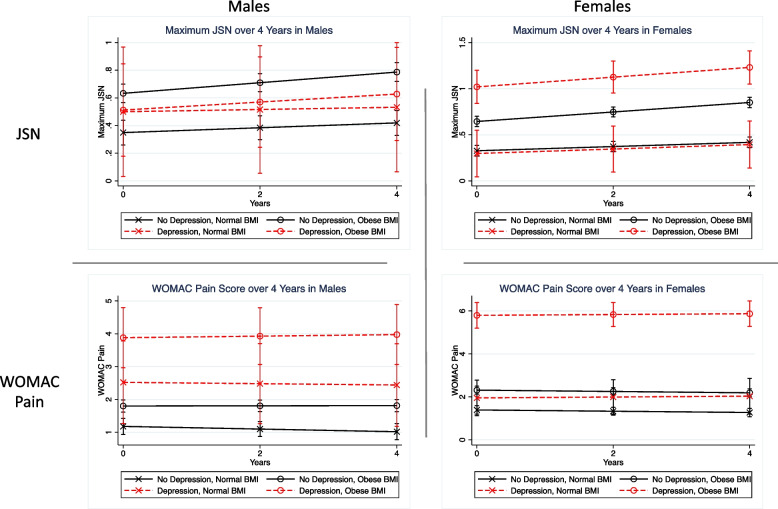
The BMI-depression-sex interactions were significant for WOMAC pain (*p* = 0.02) and JSN (*p* = 0.03); thus, each respective analysis was subdivided by males and females. For WOMAC pain, the BMI-depression interaction was significant in females (*p* < 0.001) but was not significant for males (*p* = 0.33). For JSN, the BMI-depression interaction was significant in females (*p* = 0.01) but was not significant for males (*p* = 0.35). Note that the range for the JSN score was [0 to 3] and the range for WOMAC pain score was [0 to 20]

In the sensitivity analyses, the BMI-depression-sex interaction was statistically significant for WOMAC pain (*p* = 0.02) and JSN (*p* = 0.03) and was not statistically significant for cartilage T_2_ (*p* = 0.39) as demonstrated in Table 2**.** Since these BMI-depression-sex interactions were significant for WOMAC pain and JSN, each respective analysis was subdivided by males and females **(**Fig. 3**)**. For WOMAC pain, the BMI-depression interaction was significant in females (*p* < 0.001) but was not significant for males (*p* = 0.33). For JSN, the BMI-depression interaction was significant in females (*p* = 0.01) but was not significant for males (*p* = 0.35).

### Sensitivity analysis: KL 0/1

Of all the participants included in this study, *n* = 865 had KL grade 0/1 in both knees (of those, 17 had sustained depressive symptoms and obese BMI (30–49 kg/m^2^), 17 had sustained depressive symptoms and normal BMI (16.9–24.9 kg/m^2^), 340 had no sustained depressive symptoms and obese BMI (30–49 kg/m^2^) and 491 had no sustained depressive symptoms and normal BMI (16.9–24.9 kg/m^2^). In this subset of participants with KL grade 0/1 in both knees, the BMI-depression interaction was statistically significant for WOMAC pain (*p* < 0.001 and JSN (*p* = 0.02), while it was not statistically significant for cartilage T_2_ (*p* = 0.25), Table 2**.** The significant associations found with WOMAC pain outcomes were also found in the primary analysis; however, in this subgroup analysis, JSN outcomes were also statistically significant. These results demonstrate that even in individuals without evidence of radiographic OA (KL 0/1), having sustained depressive symptoms and obesity is associated with joint structure endpoints of increased JSN, as well as increased pain over 4 years.

## Discussion

In this study, BMI-depression interactions were significantly associated with greater WOMAC knee pain in all participants, as well as greater JSN in women and participants with KL 0/1 (exploratory analysis) over 4-years. For cartilage T_2_, the group-based analysis exhibited that individuals with depressive symptoms and obesity had significant elevations in T_2_ compared to all other groups including individuals without depressive symptoms and without obesity. These results suggest that the compound effects of depression and obesity have greater impact on knee pain and JSN progression compared to what would be expected based on their *individual* effects. Thus, obese individuals with comorbid depression are likely to have worse OA outcomes over 4 years than would be predicted based on the individual effects of depression and obesity.

While many studies have reported the individual effects of both obesity and depression on OA including increased joint pain and disability [6, 9, 10] and increased radiographic degeneration [7, 10], few studies have assessed their combined impact. One study [12], however, reported that patients with obesity and comorbid depression have increased biomarkers of cartilage degradation and bony remodeling as well as worse pain and function over 2 years compared to non-obese individuals and individuals without depression. The results of our study are in agreement with, and complementary to, the results reported by Jacobs et al. [12]: both studies report increased knee pain in participants with obesity and depression, and our study further demonstrates increased JSN in a subset of participants (KL 0/1 and females) over 4 years. Collectively, these studies suggest that individuals with comorbid obesity and depressive symptoms have greater progression of symptomatic OA compared to what would be expected based on their *individual* effects.

In addition to the interaction analysis, a further examination of the group-based results is valuable to better understand the effects comorbid obesity and depressive symptoms on OA outcomes. Summarizing the interaction results: the BMI-depression interaction was significant for WOMAC pain (*p* < 0.001), while the interaction effect for JSN was *p* = 0.08 and the interaction effect for cartilage T_2_ was *p* = 0.27. Figure 2, which graphically illustrates interactive effects for all outcomes, suggests that there may be a significant interaction observable with cartilage T_2_ in a larger sample size especially since interaction analyses are often imprecise [34]. The group-based differences for cartilage T_2_ are statistically significant (as described in the results section), and thus support an association between comorbid depression-obesity and cartilage T_2_. Thus, while the interaction analysis for T_2_ outcomes was not statistically significant, further studies with larger sample sizes may detect significant associations with comorbid depressive symptoms and obesity.

The results of the sensitivity analyses (exploratory) were consistent with the results in the entire cohort; however, additional significant associations were established in individuals without evidence of radiographic OA, and gender differences were also noted. Of interest, the interaction between depressive symptoms and BMI was significant for JSN outcomes in individuals with KL 0/1 in both knees. These results suggest that despite no evidence of radiographic knee OA, individuals with an obese BMI and depressive symptoms had not only increased knee pain, but also increased JSN loss over 4 years. In the sensitivity analysis subdivided by sex, females with depressive symptoms and obesity were more likely than males to have progression of JSN and knee pain over 4 years. These results may be attributed to evidence that depressive symptoms are more common in women than men [35], and obesity is more common in women than men [36]. In addition, in women, higher Q angles increase joint malalignment and can accelerate loss of cartilage in obese individuals with knee OA [37]. Overall, the severity of radiographic OA and sex may impact the effects of depressive symptoms and obesity on OA outcomes; these are important factors to consider when designing future prospective studies.

The mechanisms responsible for the interrelationships between the comorbid obesity-depression and OA may potentially be related to increased mechanical loading and increased systemic inflammation. Obesity causes increased mechanical loading in the joint including increased compression and external adduction moments during the stance phase of gait, which have been linked to increased bone marrow lesions [38]. Obesity is also associated with increased metabolic inflammation associated with excess adipose tissue and lipids: adipose tissue secretes inflammatory mediators including cytokines and adipokines, creating a systemic environment of increased inflammation, that may lead to OA [39]. In addition to systemic inflammation, localized knee synovitis is associated with obesity, and has been linked to increased cartilage compositional degeneration, joint structure degeneration, and pain [40]. Also, depressive symptoms are associated with systemic inflammation [41], and systemic inflammation creates “a physiological environment that promotes the development of additional inflammatory comorbidities [12]” such as OA. Jacobs et al. reported that cartilage degradation and bone remodeling was evident in a subset of obese patients with comorbid depression, perhaps due to increased inflammation [12]. In addition, several studies have confirmed “the involvement of inflammation, neurotransmitters, the hypothalamic-pituitary adrenal axis, and cortisol levels in the biological mechanisms of OA and depression [41]” and a genetic component has also been proposed [42]. Overall, we hypothesize that comorbid obesity and depressive symptoms may impact symptomatic knee OA through disrupted mechanical loading patterns and through increased systemic and localized inflammation.

Understanding the interrelationships between obesity, depression and OA will help develop treatment strategies to slow progression of OA. One such potential treatment may be increased physical activity. Since physical activity levels are lower in individuals with both obesity and depression [43], and a lack of physical activity is independently associated with increased inflammation [44], exercise may be a viable treatment option for OA in patients with both obesity and depression. Exercise causes cyclic physiologic mechanical loading and unloading, resulting in anti-inflammatory effects on both systemic and local tissue levels (particularly in adipose tissue and cartilage [43]). In addition, sustained exercise is often prescribed for weight loss [45], with long term decreases mechanical loads on the knee joint. Ultimately, exercise is associated with not only decreases in metabolic and localized inflammation [46] but also decreases in the mechanical burden on joint tissue. Thereby, increased physical activity is potentially a viable treatment for patients with comorbid depression, obesity, and OA.

The primary limitations of this study are its retrospective nature, and the small sample size of participants with sustained depression. While it would be optimal to study a greater number of participants with depression, we analyzed all participants in the OAI that met the requirements of the inclusion/exclusion criteria for this study. In addition, the reasons for a participant’s obesity or sustained depression were unknown (no data available in the OAI) and the mechanisms responsible for the associations between depression and joint degeneration were not studied directly; these caveats may be addressed by a future study with a prospective design. The number of statistical analyses performed may raise concerns of multiple testing; to reduce the number of comparisons, we designed the outcomes as primary or exploratory (as described in the statistical analysis section) [33]. While analyzing cartilage MRI T_1rho_ or other cartilage quantitative measures would be of interest, we were only able to analyze T_2_ measurements as only these measurements were provided by the OAI. Despite these limitations, our study also has pertinent strengths, particularly its longitudinal follow-up and quantitative outcomes.

Overall, the results of this study suggest that comorbid obesity and depressive symptoms are associated with progression of symptomatic OA, evidenced by increased knee pain and increased JSN. The compound effects of obesity and depression on OA are greater than their individual effects. Thus, concurrent treatment of obesity and depressive symptoms (potentially through increases in physical activity) may be beneficial when developing individualized non-invasive strategies aimed to slow progression of OA.

## Data Availability

The datasets generated and/or analyzed during the current study are available from the Osteoarthritis Initiative (OAI; https://nda.nih.gov/oai).

## Abbreviations

3 T: 3 Tesla
BMI: Body mass index
Coeff.: Coefficient
CES-D: Center for Epidemiological Studies Depression Scale
DESS: 3D dual-echo in steady state
JSN: Joint space narrowing
KL: Kellgren Lawrence
ME: Multi-echo
MRI: Magnetic resonance imaging
OA: Osteoarthritis
OAI: Osteoarthritis initiative
PASE: Physical Activity Scale for the Elderly
SE: Spin echo
TE: Echo time
TR: Relaxation time
WE: Water excitation
WOMAC: Western Ontario McMaster Universities Osteoarthritis
